# Seasonal variation in the biocontrol efficiency of bacterial wilt is driven by temperature‐mediated changes in bacterial competitive interactions

**DOI:** 10.1111/1365-2664.12873

**Published:** 2017-02-23

**Authors:** Zhong Wei, Jianfeng Huang, Tianjie Yang, Alexandre Jousset, Yangchun Xu, Qirong Shen, Ville‐Petri Friman

**Affiliations:** ^1^ Jiangsu Provincial Key Lab for Organic Solid Waste Utilization Jiangsu Collaborative Innovation Centre for Solid Organic Waste Resource Utilization National Engineering Research Centre for Organic‐based Fertilizers Nanjing Agricultural University Weigang 1 Nanjing 210095 China; ^2^ Institute of Agricultural Resources and Environment Guangdong Academy of Agricultural Sciences/Guangdong Key Laboratory of Nutrient Cycling and Farmland Conservation Guangzhou 510640 China; ^3^ Institute for Environmental Biology, Ecology & Biodiversity Utrecht University Padualaan 8 3584CH Utrecht The Netherlands; ^4^ Department of Biology University of York Wentworth Way York YO10 5DD UK

**Keywords:** bacterial wilt disease, biological control, competition, competitive interactions, environmental temperature, plant pathogens, *Ralstonia pickettii*, *Ralstonia solanacearum*, tomato

## Abstract

1. Microbe‐based biocontrol applications hold the potential to become an efficient way to control plant pathogen disease outbreaks in the future. However, their efficiency is still very variable, which could be due to their sensitivity to the abiotic environmental conditions.
2. Here, we assessed how environmental temperature variation correlates with ability of *Ralstonia pickettii*, an endophytic bacterial biocontrol agent, to suppress the *Ralstonia solanacearum* pathogen during different tomato crop seasons in China.
3. We found that suppression of the pathogen was highest when the seasonal mean temperatures were around 20 °C and rapidly decreased with increasing mean crop season temperatures. Interestingly, low levels of disease incidence did not correlate with low pathogen or high biocontrol agent absolute densities. Instead, the biocontrol to pathogen density ratio was a more important predictor of disease incidence levels between different crop seasons. To understand this mechanistically, we measured the growth and strength of competition between the biocontrol agent and the pathogen over a naturally occurring temperature gradient *in vitro*. We found that the biocontrol strain grew relatively faster at low temperature ranges, and the pathogen at high temperature ranges, and that similar to field experiments, pathogen suppression peaked at 20 °C.
4. Together, our results suggest that temperature‐mediated changes in the strength of bacterial competition could potentially explain the variable *R. solanacearum* biocontrol outcomes between different crop seasons in China.
5. *Synthesis and applications*. Our results suggest that abiotic environmental conditions, such as temperature, can affect the efficacy of biocontrol applications. Thus, in order to develop more consistent biocontrol applications in the future, we might need to find and isolate bacterial strains that can retain their functionality regardless of the changing environmental conditions.

## Abstract 2

Our results suggest that abiotic environmental conditions, such as temperature, can affect the efficacy of biocontrol applications. Thus, in order to develop more consistent biocontrol applications in the future, we might need to find and isolate bacterial strains that can retain their functionality regardless of the changing environmental conditions.

## Introduction

Increasing evidence suggests that manipulation of plant microbiomes could have beneficial effects for the plant health (Berg *et al*. 2014). For example, microbial competition can restrict pathogen growth and invasion via resource competition (Mallon *et al*. 2015; Wei *et al*. 2015b), production of antibiotics (Yu *et al*. 2002; Kinsella *et al*. 2009) or parasitism (Jones *et al*. 2007; Fujiwara *et al*. 2011). As a result, there is growing interest to harness this potential for the plant protection (Haas & Defago 2005; Ongena & Jacques 2008; Wei *et al*. 2011, 2013, 2015b). The main challenge of microbe‐based biocontrol applications still, however, is their inconsistency and high variability in the disease control outcomes (Wei *et al*. 2011, 2015a). One explanation for this is that microbe–microbe interactions are very sensitive to several abiotic and biotic factors such as environmental temperature (Jiang *et al*. 2011; Hanke *et al*. 2015), productivity (Mallon *et al*. 2015) and microbial community composition (Wei *et al*. 2015b), which could affect biocontrol outcomes by changing the strength of species interactions. Here, we specifically focused on the effect of natural environmental temperature variation on the suppression of *Ralstonia solanacearum* pathogen by an endophytic *Ralstonia pickettii* strain.

Bacterial pathogen *R. solanacearum*, which causes bacterial wilt disease, is one of the most devastating plant diseases in the tropical and subtropical regions of the world (Hayward 1991; Álvarez, Biosca & López 2010). *Ralstonia solanacearum* infects its plant hosts via roots. After successful colonization, pathogen invades the plant vascular tissues, where it multiplies and clogs the xylem ultimately killing the plant (Álvarez, Biosca & López 2010). Within the plants, pathogens must compete with endophytic microbes that naturally inhabit the plant tissues (Rosenblueth & Martinez‐Romero 2006; Reinhold‐Hurek & Hurek 2011). Several endophytes can suppress pathogen growth via resource or direct interference competition (Ramesh, Joshi & Ghanekar 2009; Oliveira, Silva & Sand 2010; Tan *et al*. 2011) and some of them could potentially able to control bacterial wilt (Bloemberg & Lugtenberg 2001; Haas & Keel 2003; Upreti & Thomas 2015). In this study, we used *R. pickettii* QL‐A6, a congeneric strain of *R. solanacearum*, as a model biocontrol endophyte (Wei *et al*. 2013). When injected into the tomato stem, *R. pickettii* QL‐A6 can out‐compete *R. solanacearum* and prevent or slow down the disease progression (Wei *et al*. 2013). However, ~10% of the plants still developed disease symptoms despite the application of *R. pickettii* biocontrol strain. One potential explanation could be that this biocontrol application is sensitive to seasonally varying environmental conditions such as temperature.

Environmental temperature changes as a function of the season, the year and the local climatic conditions and is known to affect the outcome of microbial interactions (Hanke *et al*. 2015; Zander, Bersier & Gray 2017). Previous studies have demonstrated that bacterial wilt outbreaks are closely linked with high environmental temperatures (Wei *et al*. 2011, 2015a). First, this could be due to population density effects if the high temperatures benefit the pathogen growth over the biocontrol strain growth. For example, the pathogen and biocontrol strains might have different growth optima, and thus, temperature changes could affect the strength of resource or interference competition between the strains. Second, high temperature could affect the virulence expression of the pathogen: most strains of *R. solanacearum* are only pathogenic at temperatures between 25 and 35 °C, with the exception of Race 3 strains that are able to cause disease below 20 °C (Bocsanczy *et al*. 2014). Third, changes in temperature could affect the pathogen suppression by having effects on plant immune responses (Cheng Cheng *et al*. 2013) or non‐bacterial micro‐organisms present in the microbiome (Waldrop & Firestone 2006).

Here, we concentrated on the effect of temperature on microbial interactions and bacterial wilt by conducting field and laboratory experiments in the context of tomato crop protection in China. We first set up a series of field experiments where we compared the bacterial wilt disease outcomes between plants that were treated with the biocontrol strain (plants stem injection with *R*. *pickettii* QL‐A6 strain) and plants that were treated only with water (control). We considered *R*. *pickettii* QL‐A6 as an endophytic strain as this bacterium can also live within the tomato xylem without causing any visible disease symptoms (Wei *et al*. 2013). Infection experiments took place during four different alternative crop seasons that differ considerably in their mean environmental temperatures with early‐spring and late‐autumn seasons having the lowest, and the late‐spring and early‐autumn the highest mean temperatures (Wei *et al*. 2015a). The field experiments were carried out between years 2010 and 2014 in Nanjing, China, and the disease incidence (DI) and the pathogen and biocontrol bacterial densities monitored within the tomato throughout crop seasons. To study the effect of temperature on bacterial competition directly, we also conducted a series of *in vitro* microcosm experiments where we compared the growth and competition between *R. pickettii* and *R. solanacearum* strains at naturally occurring temperature range typical for field conditions in China.

## Materials and methods

### Bacterial strains and culture conditions

We used a *R. solanacearum* QL‐Rs1115 strain (GenBank accession: GU390462) tagged with the pYC12‐mCherry plasmid as a model bacterial pathogen (Wei *et al*. 2011; Tan *et al*. 2016). The *R. pickettii* QL‐A6 biocontrol strain (GenBank accession: HQ267096) was isolated from the tomato rhizosphere (Wei *et al*. 2013). We observed that *R. pickettii* QL‐A6 biocontrol strain can live within tomato xylem as an endophyte and effectively suppress the *R. solanacearum* pathogen (Wei *et al*. 2013). Both bacterial strains were routinely cultured in CPG broth (1 g of Casamino acids per litre, 10 g of peptone per litre, 5 g of glucose per litre) or CPG agar (CPG broth with 15 g of agar per litre) media (French *et al*. 1995; Wei *et al*. 2013). These strains can be easily distinguished on the basis of colony morphology and colour by using the South Africa semi‐selective medium (SMSA‐E) (French *et al*. 1995; Wei *et al*. 2013).

### Determining the *R. pickettii* QL‐A6 strain biocontrol efficiency between different crop seasons

The field experiment study site located in the town of Qilin (118°57′E, 32°03′N; previously described in detail in Wei *et al*. (2011). Total of two crops of tomatoes (*Solanum lycopersicum*) could be grown per year at this location: one in the spring and one in the autumn. Farmers also vary in their preference to sow the tomato seeds early or late of each crop season. We assessed the *R. pickettii* QL‐A6 strain ability to control bacterial wilt of tomato during four different crop seasons: early‐ and late‐spring crop seasons and early‐ and late‐autumn crop seasons between years 2010 and 2014, as shown in Table 1. We used different plots within the same field for each crop season treatment at every year. Unfortunately, we were not able to include all crop seasons to our treatments at every year due to workload and practical limitations set by the farmers. However, the experiment was duplicated or triplicated for every crop season treatment between different years (Table 1). During every crop season, we randomly selected and marked 240 (tomato cultivar Hezuo 903) after transplantation of the crops (Table 1). Half of the plants (120) were treated with 10 μL of *R. pickettii* QL‐A6 biocontrol strain suspension (10^8^ CFU mL^−1^), and another half with sterilized water (control treatment) by stem injection method (Wei *et al*. 2013). Please note that *R. pickettii* already leads to considerable pathogen suppression at much lower density, which demonstrates the practicability of our method (Wei *et al*. 2013). The inoculation time varied from 1 to 5 weeks depending on the growth season as we wanted to infect similar sized tomato plants with every crop season (tomatoes grow faster during warm and slower during the cold crop seasons; Table 1). The rationale for this was that *R. solanacearum* disease dynamics are often linked with plant development and growth. Disease development was expressed as the DI, which denoted the percentage of wilted plants on the first day of the harvest (Table 1). The disease incidence reduction efficacy (DIR) by *R. pickettii* QL‐A6 was calculated for each crop season by using the following equation: DIR (%) = (DI of control treatment−DI of *R. pickettii* QL‐A6 treatment)/DI of control treatment × 100.

**Table 1:** The dates of tomato seedling transplantation to the field, inoculation of *Ralstonia pickettii* QL‐A6 strain by stem injection and the harvest day of tomatoes for the early‐, late‐spring (ES, LS) and early‐, late autumn (EA and LA) crop seasons between years 2010 and 2014

Year	Crop season	Transplantation date	Inoculation date	Harvest date
2010	LS	27‐Mar	10‐Apr	16‐Jun
2010	EA	29‐Jul	29‐Aug	13‐Nov
2011	ES	22‐Jan	2‐Mar	2‐May
2011	LS	2‐Apr	12‐Apr	20‐Jun
2011	EA	23‐Jul	5‐Aug	15‐Oct
2011	LA	28‐Aug	7‐Sep	7‐Dec
2012	ES	15‐Jan	5‐Mar	3‐May
2012	LS	29‐Mar	13‐Apr	15‐Jun
2012	EA	20‐Jul	2‐Aug	13‐Nov
2012	LA	3‐Sep	15‐Sep	10‐Dec
2013	LS	26‐Mar	13‐Apr	19‐Jun
2013	LA	27‐Aug	9‐Sep	3‐Dec
2014	LS	29‐Mar	10‐Apr	18‐Jun

### Measuring *R. solanacearum* and *R. pickettii* cell densities in the tomato stem

The *R. solanacearum* and *R. pickettii* densities within the tomato stems were determined as follows. For each treatment, fresh stem‐base samples were collected from five randomly selected plants at every sampling point for a total of six to seven sampling points per crop season. The surface of fresh stem‐base segments (~1·5 to 2·0 cm) was first sterilized by dipping into 95% alcohol and by flaming for 3–5 s. The efficiency of surface sterilization was confirmed by placing the treated stem‐base segments on a CPG agar plate for 5 min and incubating at 30 °C for 2 days (Wei *et al*. 2011): no visible colonies were observed after 2 days of incubation. The equal sized segments (~1 cm) were weighed, ground with a bowl chopper and mixed with 9 mL of sterilized water. The resulting sample suspension was spread on SMSA‐E medium after 10‐fold dilution series. After 3 days of incubation at 30 °C, bacterial densities were measured as the number of colony forming units per gram of stem (CFU g^−1^ fresh weight). The proportion of *R. solanacearum* and *R. pickettii* cells was determined based on difference in the colony colour and morphology (Wei *et al*. 2013).

### Measuring bacterial growth and competition *in vitro* across naturally occurring temperature range

We used *in vitro* liquid microcosms assay to measure the growth and strength of direct competition (pathogen growth reduction, PGR) between *R. pickettii* QL‐A6 biocontrol and *R. solanacearum* QL‐Rs1115 pathogen strains. Briefly, individual colonies of *R. pickettii* QL‐A6 and *R. solanacearum* QL‐Rs1115 were grown in CPG broth at 30 °C for 48 h with agitation at (170 rpm). Cells were then washed by centrifugation at 5000 g at 10 °C and resuspended in 0·85% NaCl at a density of 10^9^ cells mL^−1^. In the first assay, we measured both strains’ growth separately in five different temperatures: 15, 20, 25, 30 and 37 °C – a temperature range that covers the average temperature variation between different tomato crop seasons. All cultures were started with a starting density of 10^7^ cells mL^−1^ in 200 μL in CPG broth (96 well microtiter plates) and grown for 48 h – a long enough time for bacteria to reach carrying capacities. Each treatment was replicated for three times. In the second assay, we measured the reduction in the pathogen growth by the biocontrol strain under direct competition. Briefly, suspension of the mCherry‐tagged *R. solanacearum* QL‐Rs1115 strain with a starting density of 10^6^ cells mL^−1^ was grown alone or together with *R. pickettii* QL‐A6 strain (starting density: 10^7^ cells mL^−1^) in the same temperature treatments as in the first assay. Total bacterial growth was recorded after 48 h on the basis of optical density (OD_600_), and the pathogen growth estimated on the basis of the mCherry fluorescence signal (excitation: 587 nm, emission: 610 nm) using a SpectraMax M5 Plate reader (Molecular Devices, Sunnyvale, CA, USA). PGR was calculated as the difference between the pathogen‐biocontrol and pathogen‐only treatments as follows: PGR (%) = (RFU of control−RFU of cocultures)/RFU of control × 100, where the RFU denotes for the relative fluorescence density (fluorescence signal density divided with total bacterial OD_600_ value). It has been previously shown that *R. solanacearum* virulence is affected by the environmental temperature. Hence, we also explored if the temperature affects the transition from virulent (large, pink and fluidal) to non‐virulent (small, dark red and non‐fluidal) colony morphotype by growing initially virulent *R. solanacearum* strain (QL‐Rs1115) at 4, 10, 15, 20, 25, 30 and 37 °C for 5 days until bacteria reached stationary phase (CPG broth, initial bacterial density of 10^6^ CFU mL^−1^ with six replicates per temperature treatment). Changes in colony morphology were determined by culturing subsamples from all populations on TZC agar plates.

### Statistical analysis

Daily maximum temperatures for the early‐spring, late‐spring, early‐autumn and late‐autumn crop seasons between years 2010 and 2014 (Table 1) were obtained from Weather Online (http://www.weatheronline.co.uk/). The MT_dpi_, representing the mean maximum temperature averaged over each crop season, was used to illustrate the effect of environmental temperature on bacterial growth, competition and disease development and control. The bacterial densities in the tomato plant stems were analysed by calculating the area under the curve (based on six to seven sampling time points) for both the *R. solanacearum* (MP_Rs_) and *R. pickettii* (MP_Rp_) strains. The MP_Rs_ and MP_Rp_ values were calculated independently for each crop season by using the audpc function in the R package {agricolae} and normalized with the total number of sampling time (days) to account for the differences between crop seasons. We used following equation:

$$MP_{x}=\left(\sum_{i=1}^{i=n-1}0\cdot 5\left(p_{i+1}+p_{i}\right)\left(t_{i+1}-t_{i}\right)\right)/d$$

Here, MP_*x*_ equals MP_Rs_ or MP_Rp_, *t*_*i*_ is the date we collected samples, *p*_*i*_ is the bacterial population on the date of sampling, *n* is the number of times we collected samples and *d* is the period of days between *R. pickettii* QL‐A6 application and DI recording (Table 1). We used linear models to assess the effect of crop season (factor, four levels) and environmental temperature (MT_dpi_, continuous variable) on MP_Rs_ or MP_Rp_. Similarly, linear models were used to analyse all *in vitro* microcosm data. The relative red fluorescence density and bacterial tomato stem‐base density data were log10 transformed before the analyses.

## Results

### Changes in yearly environmental air temperatures between different crop seasons

The recorded daily maximum air temperature variation between 2010 and 2014 at Nanjing (nearest city to the Qilin) is depicted (see Fig. S1a, Supporting Information). Typically, the mean monthly maximum air temperatures reached 20 °C between April and October and exceeded over 30 °C between June and August (see Fig. S1a). As a result, the mean maximum temperatures were highest for late‐spring and early‐autumn crop seasons: the MT_dpi_ of the late‐spring and early‐autumn crop seasons were above 25 °C and were significantly higher than those in early‐spring and late‐autumn crop seasons (*F*_3,9_ = 18·9, *P* < 0·001, see Fig. S1b).

### The bacterial wilt DI and *R. pickettii* biocontrol efficacy between different crop seasons

**Figure 1:**
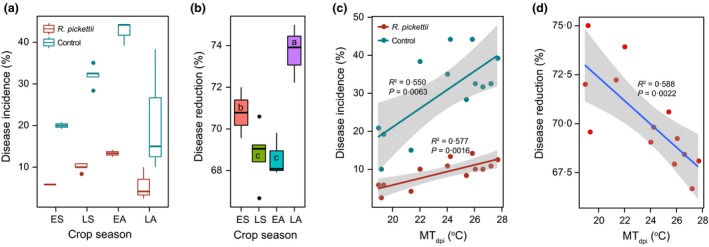
The bacterial wilt disease incidence and *Ralstonia pickettii* QL‐A6 biocontrol efficacy between different crop seasons. (a) The disease incidence (percentage of wilted tomato plants) between *R. pickettii* and control (sterilized water) treatments between different crop seasons (averaged over the years). (b) The disease reduction by *R. pickettii* QL‐A6 strain compared to control treatment between different crop seasons (averaged over the years; different letters show significant differences based on Duncan's multiple range test, *P *< 0·05). (c) Fitted linear regression between disease incidence and environmental temperature in *R. pickettii* and control treatments (mean temperatures averaged over the crop season after *R. pickettii* inoculation, MT_dpi_). (d) Fitted linear regression between disease reduction and environmental temperature averaged over the crop seasons and years. In (a) and (b), ES, LS, EA and LA denote for early‐spring, late‐spring, early‐autumn and late‐autumn crop seasons, respectively. [Colour figure can be viewed at wileyonlinelibrary.com]

We found that DI was significantly lower in the biocontrol treatment where the plants were injected with *R. pickettii* QL‐A6 strain compared to the control treatment where the plants were injected with sterile water (Treatment: *F*_1,24_ = 43·5, *P *< 0·001, Fig. 1a). Also, the crop season had a significant main effect on the DI in both the biocontrol (*F*_3,9_ = 9·6, *P *= 0·004) and control (*F*_3,9_ = 5·6, *P* = 0·019) treatments (Fig. 1a). The highest DI (percentage of wilted plants) was observed during the early‐autumn crop season, followed by the late‐spring crop season (Fig. 1a). The reduction in DI between biocontrol and control treatment varied from 65 to 75% between different crop seasons (Fig. 1b). The highest reduction was observed during the late‐autumn crop season, followed by early‐spring, late‐spring and early‐autumn crop seasons (Fig. 1b, *F*_3,9_ = 9·6, *P *= 0·004). Additional analyses showed that DI had more linear relationship with temperature in the control (Slope = 2·454, *F*_1,11_ = 11·3, *P* = 0·006) compared to the biocontrol treatment (Slope = 0·885, *F*_1,11_ = 17·4, *P* = 0·002, Fig. 1c), and that the disease reduction was clearly negatively correlated with the temperature (Fig. 1d, *F*_1,11_ = 15·7, *P* = 0·002). Together, these results suggest that while the application of *R. pickettii* QL‐A6 considerably reduced the bacterial wilt DI in general, this effect was weaker during the warmest late‐spring and early‐autumn crop seasons.

### The effect of environmental temperature and crop seasons on within‐host bacterial competition *in vivo*

**Figure 2:**
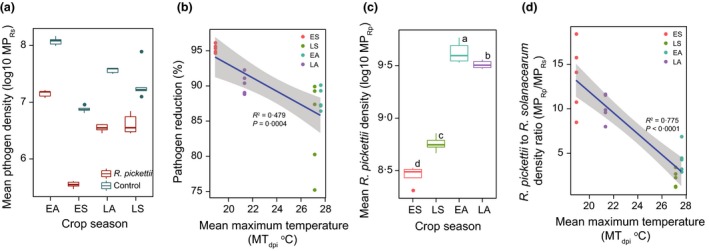
The effect of environmental temperature and crop seasons on within‐host bacterial competition *in vivo*. (a) The pathogen densities (MP_Rs_) in the tomato plant stems in *Ralstonia pickettii* and control treatments during different crop seasons. (b) The reduction in pathogen densities by *R. pickettii* during different crop seasons and across mean environmental temperature variation. (c) The *R. pickettii* densities (MP_Rp_) during different crop seasons. (d) The *R. pickettii* to *Ralstonia solanacearum* density ratio (MP_R__p_/MP_R__s_) across mean environmental temperature variation. In all panels, ES, LS, EA and LA denote for early‐spring, late‐spring, early‐autumn and late‐autumn crop seasons, respectively. In panels (a), (c) and (d), the MP_R__s_ and MP_R__p_ values were calculated independently for each crop season by using the audpc function in the R package {agricolae} and normalized with the total number of sampling time (days) to account for the differences between the crop seasons. [Colour figure can be viewed at wileyonlinelibrary.com]

**Figure 3:**
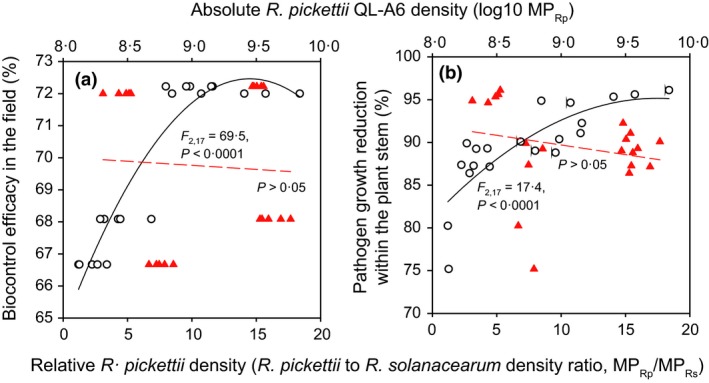
The relationship between absolute (log 10 MP_R__p_, red triangles) and relative *Ralstonia pickettii* densities (*R. pickettii* to pathogen density ratio, white circles, MP_R__p_/MP_R__s_) with biocontrol efficacy in the field (a) and pathogen growth reduction within the tomato stems (b). The black lines show a fit between relative and red dashed lines a fit with absolute *R. pickettii* densities. In (a) and (b), the MP_R__s_ and MP_R__p_ values were calculated independently for each crop season by using the audpc function in the R package {agricolae} and normalized with the total number of sampling time (days) to account for the differences between the crop seasons. [Colour figure can be viewed at wileyonlinelibrary.com]

The *R. solanacearum* densities within the tomato stems were 75–95% lower in the *R. pickettii* compared to control treatments during all crop seasons (Treatment: *F*_1,39_ = 34·2, *P *< 0·001, Fig. 2a), and the pathogen density reduction by *R. pickettii* was significantly attenuated by the increasing temperature (*F*_1,18_ = 18·5, *P *< 0·001, Fig. 2b). While the pathogen densities correlated positively with increasing temperature in both *R. pickettii* QL‐A6 (*F*_1,18_ = 45·4, *P *< 0·001) and control (*F*_1,18_ = 14·2, *P* = 0·001) treatments (data not shown), absolute pathogen numbers did not correlate linearly with DI. For example, the pathogen densities were higher during the early‐spring compared to the late‐spring season, even though the DI was higher during the late‐spring season (Figs 1a and 2a). Similarly, *R. pickettii* biocontrol strain densities were the highest during the early‐autumn season also when the DI levels peaked (*F*_3,16_ = 266·4, *P *< 0·001; Figs 1a and 2c). Crucially, we found that biocontrol agent to pathogen density ratio was clearly lowest during the relatively coldest early‐spring and late‐autumn crop seasons and that this ratio significantly decreased with the increasing temperature (population ratio; Fig. 2d, *F*_1,18_ = 66·5, *P *< 0·001). As a result, the relative biocontrol agent to pathogen density ratio predicted the biocontrol efficacy better compared to absolute biocontrol agent densities (Fig. 3). Together, these results suggest that *R. pickettii* potentially had a competitive advantage at lower, and the pathogen at higher temperature ranges leading to relatively higher biocontrol agent to pathogen density ratios during the relatively colder early‐spring and late‐autumn crop seasons.

### The effect of temperature on *R. pickettii* and *R. solanacearum* growth and competition *in vitro*

**Figure 4:**
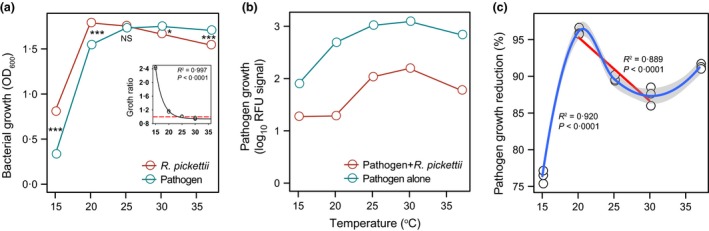
The effect of temperature on *Ralstonia pickettii* and *Ralstonia solanacearum* growth and competition *in vitro*. (a) The growth of both bacterial strains in monocultures along the temperature gradient (small panel shows the nonlinear regression fit for the growth ratio of strains: higher the value, greater the advantage of the *R. pickettii* biocontrol strain). (b) The pathogen growth in the absence and presence of *R. pickettii* biocontrol strain along the temperature gradient; pathogen growth was estimated as the log_10_‐transformed relative red fluorescence signal (denoted as log_10_ RFU signal). (c) The growth reduction of the pathogen by *R. pickettii* biocontrol strain along the temperature gradient (a fitted cubic regression; linear reduction of pathogen density between 20 and 30 °C is highlighted with a red line). In all panels, the error bars are smaller than the symbols and hence inseparable. In panel (a), asterisks * and *** denote statistically significant differences at levels of 0·05 and 0·001, respectively, between *R. pickettii* and pathogen treatments, and NS denotes for non‐significant difference. [Colour figure can be viewed at wileyonlinelibrary.com]

To causally show that temperature can change the strength of competitive interaction between the biocontrol strain and the pathogen, we compared the strains’ growth in mono‐ and co‐cultures in microcosm experiments. We found that temperature changed the growth of both *R. pickettii* and *R. solanacearum* strains when measured in monocultures (Fig. 4a). In general, *R. pickettii* grew significantly better at lower temperatures (15 and 20 °C: one‐way ANOVA, *F*_1,4_ = 5841, *P *< 0·001 and *F*_1,4_ = 123·9, *P* < 0·001, respectively), while *R. solanacearum* grew significantly better at higher temperatures (30 and 37 °C: one way ANOVA, *F*_1,4_ = 11·1, *P *< 0·029 and *F*_1,4_ = 1614, *P *< 0·001, respectively). No significant difference was observed at 25 °C (one‐way ANOVA, *F*_1,4_ = 1·5, *P *= 0·295). Thus, the ratio of growth between *R. pickettii* and the pathogen decreased sharply with increasing temperature from 15 to 20 °C, and then decreased slowly with increasing temperature (the small panel in Fig. 4a, nonlinear regression with exponential decay model, *F*_1,13_ = 141·1, *P *< 0·001). The change from virulent to avirulent colony morphology occurred only when initially virulent *R. solanacearum* strain was incubated at 37 or 4 °C (Fig. S2).

When cultured together, pathogen density was significantly reduced by *R. pickettii* (Fig. 4b: *F*_1,28_ = 42·3, *P *< 0·001; estimated as the relative red fluorescence signal intensity). The reduction in pathogen growth was highest at 20 °C (*F*_4,10_ = 269·7, *P *< 0·001, Fig. 4c), which declined from this point on with increasing temperature (Fig. 4c, red line, linear regression, *R* ^2^ = 0·889, *P *< 0·001). These results show that the temperature can change the strength of competitive interaction between the pathogen and the biocontrol strain.

## Discussion

Here, we studied how seasonal temperature variation affects the efficacy of endophytic *R. pickettii* biocontrol agent in China. We found that bacterial wilt DI varied between crop seasons having highest levels of disease during the relatively warmest late‐spring and early‐autumn crop seasons. Unexpectedly, low levels of DI did not correlate with low absolute pathogen densities or high absolute biocontrol agent densities within the tomato plants. Instead, high *R. pickettii* to *R. solanacearum* density ratio was a more important predictor of DI and pathogen suppression within the plant xylem. Mechanistically, this could be explained by temperature‐mediated changes in bacterial competitive interactions, where *R. pickettii* biocontrol agent was able to out‐compete the pathogen at low‐temperature range, while the pathogen was able to out‐compete the biocontrol agent at high‐temperature range. Together, these results suggest that temperature variation could play a key role in determining bacterial wilt disease outbreaks by changing the competitive interactions between the invading pathogen and the defending biocontrol agent.

The level of bacterial wilt disease was the highest, and the level of disease reduction the lowest during the warmest crop seasons. This result is in line with a previous study where *Bacillus amyloliquefaciens* biocontrol agent failed to control bacterial wilt disease during the relatively warm late‐spring and early‐autumn crop seasons (Wei *et al*. 2011, 2015a,b). Nevertheless, while Wei *et al*. 2015a,b found that high levels of DI coincided with high absolute pathogen densities in the rhizosphere and within the tomato stem, we found that high levels of DI did not coincide with high absolute pathogen densities within the tomato stems. Instead, the highest absolute pathogen densities were observed during the relatively cold early‐spring crop season with relatively low levels of bacterial wilt disease. Similarly, the high biocontrol agent densities did not coincide with crop seasons with the highest levels of disease reduction. However, the biocontrol agent to pathogen density ratios were clearly lowest during the late‐spring and early‐autumn crop seasons when the levels of DI were also the highest. This suggests that the relative biocontrol agent density was a more important predictor of the bacterial wilt disease dynamics than the absolute biocontrol agent density. From diagnostics point of view, monitoring changes in the relative pathogen densities could thus be a better way to predict the bacterial wilt disease outbreaks.

Variation in environmental temperature between crop seasons was an important driver of the disease outcome by having an effect on biocontrol agent to pathogen density ratio. Temperature variation could, for example, change the bacterial competitive interactions by shifting the growth optima of one or both species. In support for this, we found that the biocontrol agent was able to grow better at low and the pathogen at high‐temperature range *in vitro*, while the crop seasons with low DI were characterized by high biocontrol agent to pathogen density ratios in our field experiments. *Ralstonia solanacearum* strains originating from tropical areas have generally a high‐temperature optimum (35 °C), whereas the strains occurring at higher altitudes in the tropics, in subtropical and temperate areas have lower temperature optimum (27 °C) (EPPO 2004). While *R. pickettii* is also able to grow at 35 °C temperature, it can also grow well at low temperatures (Labarca *et al*. 1999). As a result, bacterial strains that can retain their functionality across varied environmental conditions could thus be good candidate species for developing more consistent biocontrol applications. In the case of bacterial wilt, biocontrol species that can out‐compete *R. solanacearum* during the warm crop seasons would especially be useful. Alternatively, biocontrol strains with different temperature optimums could be used during different tomato crop seasons: one effective in colder and the other in the warmer climate.

Temperature variation could have also affected bacterial wilt disease dynamics indirectly via effects on *R. solanacearum* virulence gene expression or by affecting the tomato plant immune responses. First, the high environmental temperature could have increased *R. solanacearum* virulence via density‐dependent virulence gene expression mediated by bacterial cell‐to‐cell signalling (i.e. quorum sensing) (von Bodman, Bauer & Coplin 2003). High temperature (32 °C) has also been found to directly increase the severity of bacterial wilt in two tomato lines (Philippine 1169 and Hawaii 7580) (Krausz & Thurston 1975). In contrast, low soil temperatures could reduce disease development by directly inducing tomato resistance (Mew & Ho 1977) or by attenuating *R. solanacearum* virulence via lowered or lost twitching motility – a trait important for plant root colonization and invasion (Kang *et al*. 2002; Bocsanczy *et al*. 2014). While these hypotheses remain to be tested in future experiments, our preliminary data suggest that only very cold temperatures (around 4 °C) and very hot temperatures (around 37 °C) lead to the emergence of small *R. solanacearum* colony variants indicative of lowered pathogen virulence (see Fig. S2). As a result, temperature‐mediated effects played likely only a minor role for the biocontrol outcomes and pathogen virulence in our experiments: environmental temperatures ranged from 10 to 35 °C most of the crop seasons (see Fig. S1). In general, our results support the idea that environmental temperature has significant role for the severity of bacterial wilt infections, and hence, shifting tomato plant transplantation regime to avoid high‐temperature periods could be a simple and efficient way to enhance biocontrol of bacterial wilt (Wei *et al*. 2015a).

Competition between the pathogenic and non‐pathogenic bacteria is a major constraint for disease outbreaks in plant microbiomes (Hanemian *et al*. 2013; Wei *et al*. 2013; Raaijmakers & Mazzola 2016). The outcome of microbial competition is however very variable and often depends on abiotic environmental conditions (Jiang *et al*. 2011; Hanke *et al*. 2015), the survival of the biocontrol agent (Hu *et al*. 2016) and the interactive effects within microbiomes (Thomas & Sekhar 2016). To develop more consistent biocontrol applications, effort should be put on studying how seasonal and spatial variation in abiotic and biotic soil properties affect the stability and strength of biocontrol agent–pathogen species interactions. For example, biocontrol outcomes could vary between different fields depending on the soil type and the given microbial community composition, which could considerably affect the biocontrol agent survival (Hu *et al*. 2016) and antimicrobial activity in the vicinity of the target host (Tyc *et al*. 2014). Even though recent studies suggest that simple laboratory experiments can predict disease dynamics in the rhizosphere (Wei *et al*. 2015b; Hu *et al*. 2016), more research is still needed to quantify the strength of microbial competition in more natural conditions (e.g. in soil microcosms) (Gomez & Buckling 2013). Interestingly, even though the crops season had a clear effect on the biocontrol outcomes, the seasonal variation in DI and disease reduction were both less than <10% in *R. pickettii* biocontrol treatment in the field experiments (Fig. 1a and b). Such a relatively small variation suggests that *R. pickettii* QL‐A6 has the potential to perform consistently under temporally varying environments, which would make it ideal biocontrol agent for naturally varying field conditions.

## Authors' contributions

Z.W., Q.R.S. and Y.C.X. conceived the ideas and designed methodology; Z.W., T.J.Y., and J.F.H. collected the data; Z.W. and V.P.F. analysed the data; Z.W., J.F.H., A.J. and V.P.F. led the writing of the manuscript. All authors contributed critically to the drafts and gave final approval for publication.

## Data accessibility

Data are available from the Dryad Digital Repository https://doi.org/10.5061/dryad.50584 (Wei *et al*. 2017).

## Supporting information


**Fig. S1**. Annual environmental temperature variation between different crop seasons.

**Fig. S2**. Colony morphology of *Ralstonia solanacearum* strain QL‐Rs1115 when grown across different temperatures on TZC agar plates.
