# Supplementary material for: Seasonal variation in the biocontrol efficiency of bacterial wilt is driven by temperature‐mediated changes in bacterial competitive interactions
Source: J Appl Ecol. 2017 Feb 23;54(5):1440–8. doi: 10.1111/1365-2664.12873 (PMC5638076; doi:10.1111/1365-2664.12873)
Supplement: Supplementary file 1 — Fig. S1. Annual environmental temperature variation between different crop seasons. [file JPE-54-1440-s001.docx]

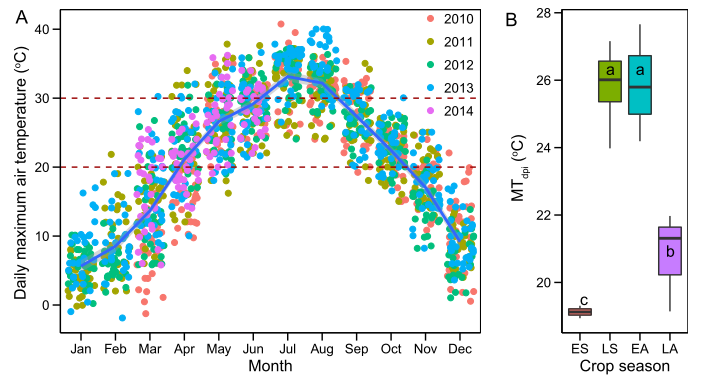


**Figure S1. Annual environmental temperature variation between different crop seasons.** The panel A shows changes in the daily maximum temperatures between years 2010 and 2014. The panel B shows the mean maximum temperatures for different crop seasons averaged over the years after *R. pickettii* QL-A6 inoculation (MT_dpi_). In panel B, ES, LS, EA and LA denote for early-spring, late-spring, early-autumn and late-autumn crop seasons, respectively (detailed information on each crop season is presented in Table S1); different letters show significant differences between crop seasons (Duncan’s multiple range test, *P* < 0.05).
