# Supplementary material for: Seasonal variation in the biocontrol efficiency of bacterial wilt is driven by temperature‐mediated changes in bacterial competitive interactions
Source: J Appl Ecol. 2017 Feb 23;54(5):1440–8. doi: 10.1111/1365-2664.12873 (PMC5638076; doi:10.1111/1365-2664.12873)
Supplement: Supplementary file 2 — Fig. S2. Colony morphology of Ralstonia solanacearum strain QL‐Rs1115 when grown across different temperatures on TZC agar plates. [file JPE-54-1440-s002.docx]

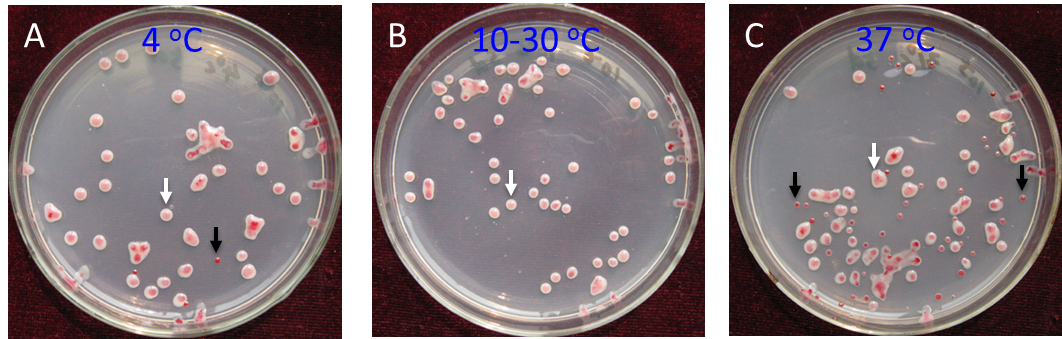


Figure S2. **Colony morphology of *Ralstonia solanacearum* strain QL-Rs1115 when grown across different temperatures on TZC agar plates**. Virulent *R. solanacearum* strain QL-Rs1115 was inoculated into CPG broth at an initial concentration of 10^6^ cfu/ml, and then cultured at 4 °C (A), 10 °C, 15 °C, 20 °C, 25 °C, 30 °C (B) and 37 °C (C) for 5 days (N=6). The change from virulent (large, pink and fluidal) to avirulent (small, dark red and non-fluidal) colony morphology of *R. solanacearum* when incubated at 37 °C or 4 °C.
